# Analysis and classification of droplet characteristics from atomizers using multifractal analysis

**DOI:** 10.1038/s41598-019-52596-6

**Published:** 2019-11-07

**Authors:** V. Godavarthi, K. Dhivyaraja, R. I. Sujith, M. V. Panchagnula

**Affiliations:** 1 Department of Aerospace Engineering, Indian Institute of Technology Madras, Chennai, 60036 India; 2 Department of Applied Mechanics, Indian Institute of Technology Madras, Chennai, 600036 India

**Keywords:** Engineering, Fluid dynamics

## Abstract

Atomizers find applications in diverse fields such as agriculture, pharmaceutics and combustion. Among the most commonly found atomizer classes of designs are pressure swirl, airblast and ultrasonic atomizers. However, it has thus far not been possible to identify the class of an atomizer from spray characteristics. We perform multifractal detrended fluctuation analysis on the droplet inter-arrival times, diameters and axial velocities of pressure swirl, airblast and ultrasonic nebulizer sprays to quantify the differences in complexity in the respective signals. We show that the width of the multifractal spectrum of the signals of droplet diameters and the inter-arrival times, measured at the edge of the spray are robust atomizer identifiers. Further, we show the presence of correlations among the droplet diameters which are otherwise considered as random or derived from a log-normal distribution. This study can be further generalized to classify fluid mechanical systems or biological sprays using an appropriately chosen single point measurement in the flow field.

## Introduction

Fluid mechanical systems are some of the most complex engineering systems in the world. They exhibit inherent nonlinearity and as a result, multiple states of operation. Computational modeling of such systems has evolved to be able to capture the flow physics through tools such as Direct Numerical Simulation. However, the inverse problem of inferring information pertaining to the flow geometry, or even the class of flows when sparse flow field measurements are given has not even been attempted in a general sense. We show in a model multiphase flow system that such identification is not only possible but that it is robust and can be generalized with just a single appropriately chosen point where flow field information is available.

We will rely on a spray injector system (an atomizer) to demonstrate the concept of identifying the source, given sparse data. Conversion of a bulk volume of liquid into droplets of various sizes, referred to as atomization, finds diverse applications in pharmaceutical, combustion, agricultural and spray painting industries. Spray characteristics from an atomizer decide the mixing efficiency, emission of pollutants, combustion efficiency and liquid vaporization times^1,2^. Hence, it is vital to study the atomization process in various atomizers.

Atomization process mainly involves three mechanisms, one in which the pressure drop across the orifice is used to breakdown the liquid sheet into droplets, another in which the kinetic energy of the flowing airstream is used to disintegrate the liquid sheet and another in which the acoustic waves are used to break the liquid into droplets. A pressure swirl atomizer (PSA) implements the first type of atomization process. Alternatively, an airblast atomizer (ABA) produces a spray due to the interaction between a high-velocity air jet and the liquid sheet^3^. For both pressure swirl and airblast atomizers swirling motion is imparted to the liquid and the air to produce a conical spray. An ultrasonic nebulizer produces an aerosol using acoustic waves generated at very high frequency to break the liquid surface into droplets. These droplets are carried by air inflow. Unlike the ABA and the PSA which generate a conical spray, a scattered aerosol spray is obtained^4^. Fundamentally, in the case of a PSA, the droplet stream (spray) imparts momentum to the concomitant air flow field. In contrast, in an ABA and an ultrasonic nebulizer, it is the air flow field that is typically the source of droplet momentum. We will show that this fluid mechanic difference manifests as difference in multifractality in droplet size, inter-arrival times (the time intervals between the arrivals of two successive droplets) and velocity measurements.

Atomization is a complex process. The spray characteristics depend on several factors such as design, mechanism, size, shape of the nozzle. Several studies have attempted to model and analyze the droplet characteristics from airblast and pressure swirl atomizers. However, a literature survey on the atomization process in PSA, ABA and ultrasonic nebulizers shows that the understanding is incomplete^3,5–7^. Studies attributed the incomplete understanding to the complexity in the atomization process, sensitive dependence of the droplet characteristics on the complex geometry of the atomizer and the inaccuracy in measurement techniques^3,8,9^.

Quantifying the complexity in the atomization process helps improve the understanding of the atomization process in different atomizers, classifying different types of atomizers and also helps in validating the models developed for various atomizers. We expect the complexity in the atomization process to reflect in the spray characteristics. In our present work, we perform multifractal analysis on the spray characteristics of the ABA, the PSA and the nebulizer. We show that this analysis can be used to classify the sprays from different atomizers and thereby to improve the current understanding of spray characteristics.

Multifractal description of a signal provides a peek into characterizing the complexity of the signal. The concept of a ‘fractal’ is introduced to describe the objects which exhibit self-similarity at different scales^10^. For fractal objects, one cannot determine measures such as length and area, as these quantities depend on the scale of resolution^11^. For example, the dimension of a straight line is 1. However, there is no integer dimension for a coastline with wrinkles, as the length of the coastline depends on the scale of magnification^11^. The dimension for such fractal curves varies between 1 and 2. The logarithmic plot of the length of the fractal curve measured with different scales will be a straight line with a negative slope, and the slope of this straight line is the fractal dimension.

Similar to a fractal object, we observe self-similarity at various time scales in a fractal time series. For a fractal time series *x*(*t*), *x*(*ct*) = *c*^*H*^*x*(*t*) is also a fractal time series with the same statistics^12^, where the scaling exponent *H* is the Hurst exponent. This scaling relationship is exploited to estimate the Hurst exponent of a signal. The lower and upper bounds for the scales are dependent upon the particular signal under consideration. In some signals, the scaling behavior is complex; the scaling exponent depends on the amplitude of fluctuations. These signals are called multifractal signals. Characterizing such signals using a single Hurst exponent is not possible^13^ and we need to determine generalized Hurst exponents for different orders.

We estimate the generalized Hurst exponents of a signal using multifractal detrended fluctuation analysis (MFDFA)^14^. We provide a brief description of MFDFA in the Methods section. The characteristics of the multifractal spectrum (the width and the generalized Hurst exponent of second order) describe the long term correlations and the complexity present in the signal. In the recent past, MFDFA has been extensively used in diverse fields such as astrophysics^15^, biomedicine^16^, engineering^17–19^, and stock markets^20^. In biomedicine, the width of the multifractal spectrum (*W*), the second order Hurst exponent (*H*) and the skewness of the spectrum (Δ*α*) are used to differentiate patients with various heart diseases, to distinguish multiple areas of the brain, identify various neuronal activities etc., to name a few^21–23^. Ali *et al*.^19^ performed MFDFA on the velocity field at various locations in a wind turbine wake, and they observed that quantities such as *H* and *W* could be used to identify various regions in the wind turbine wake. Nair and Sujith^17^ and Unni and Sujith^18^ used *H* to detect the transition between different thermoacoustic states in a turbulent combustor.

Next, we perform multifractal analysis on a range of spray characteristics viz., the droplet diameters, the axial velocities, and the inter-arrival times. The efficacy of the spray is dependent mainly on the droplet size distribution^24^. Hence, generally, the variation in the mean droplet diameters and the droplet size distributions with different operating conditions^25,26^ is studied to understand the effect of the atomizer type or dimensions on the droplet characteristics. However, the axial velocities of the droplets affect the droplet trajectories which, in turn, affect the interaction between the droplets and the target (e.g., a plant in the case of agricultural sprays or any surface in the case of spray painting applications^27^). Traditionally, axial velocities at different flow conditions are studied using the mean velocity distributions^28,29^. In addition to the droplet diameters and the axial velocities, the inter-arrival times of the droplets determine the presence of droplet clusters in the sprays.

Clustering is a phenomenon where drops are closely packed together in space. Clustering of the droplets is of primary concern in liquid-fueled combustion systems. The dispersion of droplets leads to unsteady spray behavior, which manifests as unsteadiness in the fuel flow rate in the combustion chamber^30^. The fluctuations in the fuel flow rate can cause fluctuations in equivalence ratio, which is a major cause of oscillatory instabilities in gas turbine engines^31^. Comparing the histogram of inter-arrival times with Poisson statistics^30^ or spatial imaging of the liquid flows are generally used to identify the locations of droplet clustering.

Thus, traditionally, sprays are characterized using mean quantities. A few studies performed fractal and multifractal analysis on some of the spray characteristics. Grout *et al*.^32^ calculated the fractal dimension of liquid flow images. They demonstrated that liquid atomization is a fractal process. Further, Zou & Yu^33^ performed a multifractal analysis on the droplet size distribution of an airblast atomizer. Using the multifractal spectrum, they proposed a random multifractal model to match the experiments. Both these studies demonstrate the relevance of the application of fractal concepts to the atomization process. However, a rigorous quantification is required to classify and quantify the complexity in the sprays from different atomization processes.

In general, various tools are used to analyze different spray characteristics, droplet diameters, axial velocities, and inter-arrival times. We propose a unified framework to analyze all the spray characteristics. In our present study, we analyze the droplet inter-arrival times, diameters and axial velocities of the sprays produced from a PSA, an ABA and an ultrasonic nebulizer at various axial and radial locations using MFDFA. We show that *W* derived from inter-arrival times and droplet diameters are robust discriminants. We report the relationship between *H* of the inter-arrival times of the droplets and the droplet clustering at various locations. This procedure can be generalized to other areas of fluid mechanics to classify different mechanisms or even different hardware using a single point measurement in a flow field. For instance, distinguishing patients using sprays produced while breathing or coughing could be possible.

## Results

We acquired the droplet characteristics of the sprays from the PSA the ABA, and the ultrasonic nebulizer at various axial and radial locations using a Phase Doppler Interferometer (PDI). The data for the PSA was taken from Dhivyaraja *et al*.^34^, and the data from the ABA was taken from Rayapati *et al*.^35^ The droplet characteristics include the diameters, axial velocities and the inter-arrival times.

**Figure 1:**
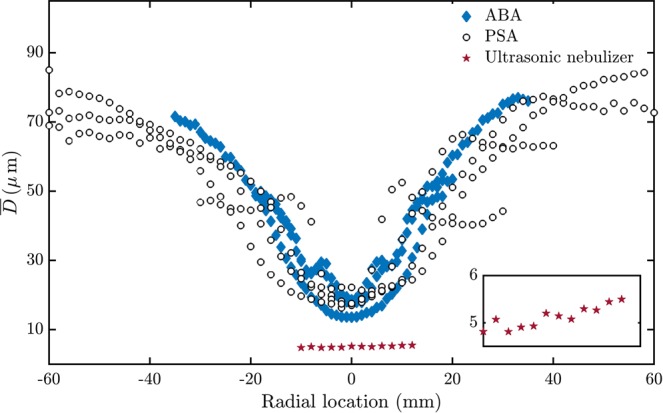
Radial distribution of mean droplet diameters of the pressure swirl, airblast and the ultrasonic nebulizer sprays. Variation of the mean diameter ($\overline{D}$) of the droplets with radial location (in mm) is shown for sprays produced from PSA, ABA and ultrasonic nebulizer. $\overline{D}$ attains a maximum near the edge of the sprays. $\overline{D}$ varies in a similar fashion for the sprays produced from ABA and PSA but is different for ultrasonic nebulizer. Hence, $\overline{D}$ cannot be used to distinguish between the sprays formed from PSA and ABA.

Traditionally, the sprays are typically characterized by their mean droplet diameters ($\overline{D}$), as shown in Fig. 1. The droplet diameter distribution of the sprays produced from PSA and ABA are similar and hence cannot be used to differentiate between the sprays produced from these atomizers. The distribution of $\overline{D}$ fails to capture the difference in the atomization mechanisms of the ABA and the PSA (Fig. 1). However, distribution of $\overline{D}$ of the ultrasonic nebulizer spray is quite different from the PSA and the ABA.

### Multifractal analysis of spray characteristics

#### Quantification of complexity using the width of multifractal spectrum (*W*)

*W* quantifies the complexity and the multifractality embedded in a signal^36^. The width indicates the range of scaling exponents required to describe the signal. A signal with a higher width is described to be more complex and multifractal than the one with a smaller width.

#### Width of multifractal spectrum of the inter-arrival times (*W*_*τ*_)

**Figure 2:**
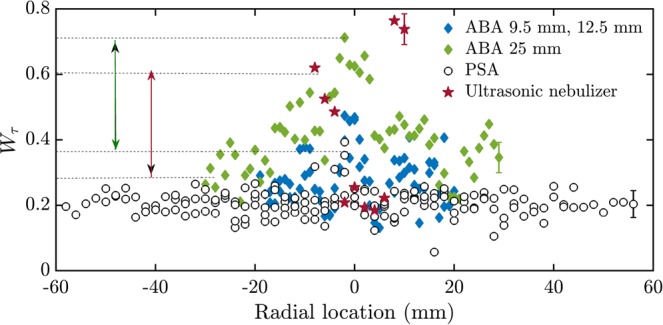
Width of the multifractal spectrum of the inter-arrival times of the pressure swirl, the airblast and the ultrasonic nebulizer sprays. The width of the multifractal spectrum (*W*_*τ*_) of the inter-arrival times of the spray are shown for axial distances of 9.5 mm, 12.5 mm and 25 mm for the ABA, at 11 mm, 22 mm, 33 mm and 44 mm for the PSA and at 5 mm for the ultrasonic nebulizer. *W*_*τ*_ of the spray formed from the ABA at an axial location of 25 mm is much higher in general than the spray produced from the PSA at all axial locations. The green arrow in the figure depicts a significant difference in the widths of the spectra corresponding to the PSA and the ABA when measured at the spray central axis towards the far field of the atomizer. The maroon arrow depicts a significant difference between the spectra corresponding to the ultrasonic nebulizer from both the ABA and the PSA when measured near the edge of the spray. A significant variation in *W*_*τ*_ with axial distance from the atomizer exit is seen for ABA. The mean error bars of different atomizers is shown at radial locations corresponding to the right edge of the sprays.

We observe that *W*_*τ*_ of the PSA does not change with axial distance and hence they are plotted together (Fig. 2). In the case of PSA, the measurements were performed at the axial locations of 11 mm, 22 mm, 33 mm, and 44 mm. On the other hand, *W*_*τ*_ of the airblast spray increases as we move farther from the injector exit and hence, they are grouped together accordingly. In the case of ABA, a strong counter-flow toroidal recirculation zone (CTRZ) leads to the presence of long-range correlations in the inter-arrival times, thereby increasing *W*_*τ*_ near the spray central axis. For the ultrasonic nebulizer, measurement is performed at an axial distance of 5 mm. *W*_*τ*_ of the ultrasonic nebulizer is maximum at the edge of the spray and minimum near the spray central axis unlike the PSA and the ABA.

There is a significant difference between *W*_*τ*_of the nebulizer spray and those of the PSA and the ABA near the edge of the spray (indicated by maroon arrow). Hence *W*_*τ*_ can distinguish the ultrasonic nebulizer near the edge of the spray. When measurement is performed close to the spray central axis, *W*_*τ*_ can differentiate between the PSA and the ABA (shown by the green arrow).

Fundamentally, the PSA, the ABA and the nebulizer are different in the nature of momentum exchange between the droplets and the surrounding gas phase. The droplets formed in an ABA spray are typically moving slower than the surrounding gas phase. In other words, the ABA spray is best described as containing droplets embedded in a turbulent swirling gas jet. The gas jet, as a result, has several embedded coherent structures which are responsible for clustering and velocity field inhomogeneity. The turbulent swirling gas jet which drives the droplets enables a high correlation. Even in the case of the ultrasonic nebulizer, the incoming air carries the droplets upwards. We can expect high complexity, thereby high *W*_*τ*_ near the edge since that is the inlet of the air. In contrast, the gas phase velocity field in a PSA spray is established due to entrainment initiated by the moving droplets. The high inertia droplets are not as affected by the surrounding air as much as in the case of the ABA or the ultrasonic nebulizer. As a result, the inter-arrival times signal of PSA is likely to be less complex.

Kantelhardt^14^ proposed two possible sources of multifractality in a signal. The first one is due to the broad probability distribution of the values in the signal. The second is the presence of long-range correlations for the different magnitudes of fluctuations in the signal. These two sources of multifractality are differentiated by computing the width of the multifractal spectrum for the randomly shuffled signal and comparing with the original one. If the randomly shuffled data retains multifractality, it is because of the presence of a broad probability density function whereas, the reduction in the width of the spectrum for the randomly shuffled data indicates the presence of long-range correlations.

**Figure 3:**
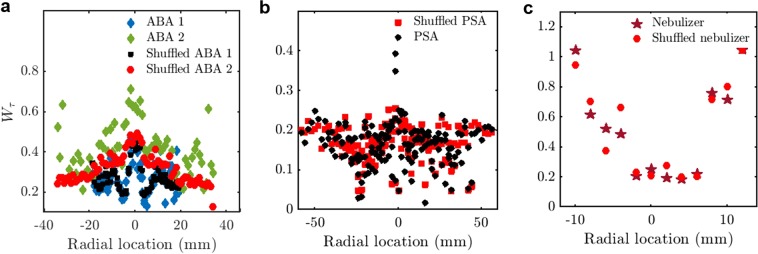
Width of the multifractal spectrum of the original and randomly shuffled inter-arrival times of the pressure swirl, the airblast and the ultrasonic nebulizer sprays. (**a**) *W*_*τ*_ for the ABA at 9.5 mm, 12.5 mm (ABA 1) and 25 mm (ABA 2) from the atomizer exit the corresponding randomly shuffled signals, Shuffled ABA 1, Shuffled ABA 2. (**b**) *W*_*τ*_ for the PSA at the axial distances of 11 mm, 22 mm, 33 mm and 44 mm from the atomizer exit and randomly shuffled signal (Shuffled PSA). (**c**) *W*_*τ*_ for the ultrasonic nebulizer the axial distance of 11 mm from the atomizer exit at various radial locations. *W*_*τ*_ of the randomly shuffled signal is less than the inter-arrival times in the case of the far field spray produced from the ABA, indicating the presence of long-range correlations.

In order to isolate the source of multifractality due to the long-range correlations among different orders of fluctuations, we compare *W*_*τ*_ with the randomly shuffled inter-arrival times (Fig. 3). We observe that *W*_*τ*_ of the randomly shuffled inter-arrival times of the airblast spray measured at an axial location of 25 mm (indicated in the legend as Shuffled ABA 2) show a significant drop from the original signal (indicated as ABA 2). This reduction in *W*_*τ*_ indicates the presence of long-range correlations among the large and small scale fluctuations in the inter-arrival times. The reduction in *W*_*τ*_ is also seen in the pressure swirl spray at the spray central axis. Nonetheless, the reduction in *W*_*τ*_ is not as significant at other axial and radial locations of the spray from the ABA (ABA1 and Shuffled ABA1), the PSA (PSA and Shuffled PSA) and the ultrasonic nebulizer (Nebulizer and Shuffled nebulizer). The resultant multifractality even after random shuffling of the data is because of the broad probability distribution of the inter-arrival times.

#### Width of multifractal spectrum of the droplet diameter signal (*W*_*D*_)

**Figure 4:**
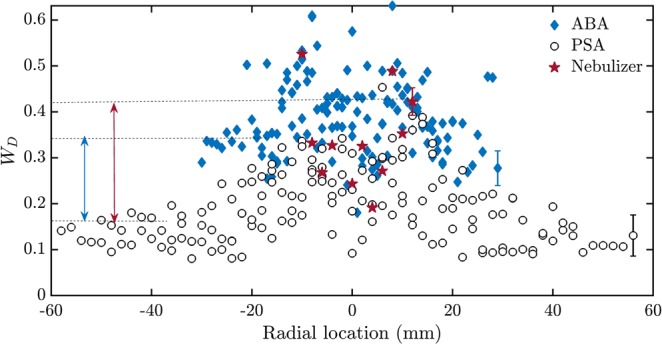
Width of the multifractal spectrum of the droplet diameter series of the pressure swirl, the airblast and the ultrasonic nebulizer sprays. Variation in the width of the multifractal spectrum of the diameter (*W*_*D*_) of the droplets acquired at different axial locations from the PSA, the ABA and the ultrasonic nebulizer with radial location is shown. The droplet diameters in the airblast spray and the ultrasonic nebulizer spray have a wider range of scaling exponents than those from the pressure swirl spray. The difference in *W*_*D*_ between the PSA and both the ABA and the ultrasonic nebulizer is maximum near the edge of the spray and hence can be used as a classifier of PSA (as indicated by the arrow). The mean error bar corresponding to an atomizer is shown on the radial location of the right edge of the sprays.

Further, we estimate *W*_*D*_ of the droplet diameters (Fig. 4). *W*_*D*_ does not vary much with the axial location and hence they are grouped together. *W*_*D*_ is higher for the droplet diameter series of the spray obtained from the ABA and the ultrasonic nebulizer than those obtained from the PSA. The difference in *W*_*D*_ is significant near the spray edge. Thus, *W*_*D*_ is a robust classifier of the PSA when measurement is performed at the edge of the spray.

**Figure 5:**
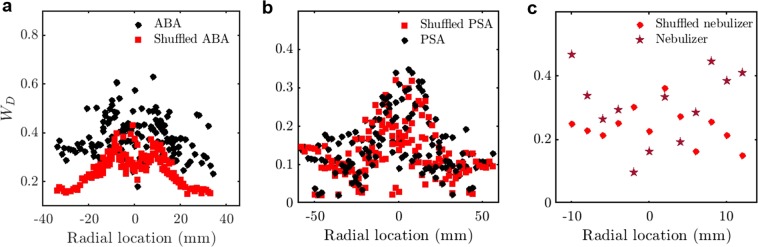
Width of the multifractal spectrum of the original and randomly shuffled droplet diameters of the pressure swirl, the airblast and the ultrasonic nebulizer sprays. (**a**) Variation of *W*_*D*_ with radial location for the ABA (**b**), for the PSA (**c**), for the ultrasonic nebulizer and their corresponding randomly shuffled signals (Shuffled ABA, Shuffled PSA and Shuffled nebulizer) measured at various axial locations. (**a**) *W*_*D*_ of the randomly shuffled diameters is significantly reduced indicating the presence of long-range correlations. (**b**) *W*_*D*_ is almost same for both the original and the randomly shuffled signal indicating only a broad probability density function, which leads to a multifractal signal. (**c**) *W*_*D*_ is reduced for the shuffled signal near the edge of the spray indicating the presence of long range correlations among the diameter sizes at the edge of the spray.

*W*_*D*_ for the shuffled data shown in Fig. 5 depends only on the width of the droplet diameter *pdf*. The diameter distributions for all the atomizers are compared in Supplementary Fig. S3. In comparison, *W*_*D*_ for the actual data is a function of the droplet diameter as well as any temporal correlation structure embedded in the data. One can observe that *W*_*D*_ for the ABA and the ultrasonic nebulizer (Fig. 5a,c) are markedly different, indicating the presence of long-range correlations among different orders of fluctuations in addition to the broad droplet diameter *pdf*. However, *W*_*D*_ is not so different for the PSA (Fig. 5b). The long-range correlations might be due to the presence of vortices in the sprays from the ABA which centrifuges like-sized droplets towards the edge of the spray, thereby causing the correlations. In contrast, the lack of strong vortical interactions precludes such significant long-range correlations in the spray from a PSA (Fig. 5b). The strong interaction between the air and the droplets near the edge of the spray in ultrasonic nebulizer cause high *W*_*D*_. In contrast to the traditional view that the droplet diameters are random^37,38^, we report the existence of correlations in the airblast spray using *W*_*D*_ and the Hurst exponent of the diameters (*H*_*D*_) (Figs 4 and 7).

In spite of the random shuffling of the diameter time series, the multifractal spectrum does not collapse into a monofractal one (*W*_*D*_ ∼ 0 for a monofractal spectrum and around 0.06 for a random noise signal depending on the realization). We conjecture that the droplet inertia is a reason for the presence of multifractality. For validation, we performed multifractal analysis on the droplet diameter series of an aerosol plume (typically produced by a medical nebulizer) where droplet inertia is extremely low. We compare $\overline{D}$ and $\overline{u}$ of the aerosol plume with those of pressure swirl, airblast and the ultrasonic nebulizer sprays near the spray central axis. The spray central axis is where $\overline{D}$ is minimum for the airblast and the pressure swirl sprays (Fig. 1).

For the aerosol plume with an air supply pressure of 50 psi, $\overline{D}=2\,\mu m$, $\overline{u}=2$ m/s. Similarly, for the airblast spray in our study, $\overline{D}=18\,\mu m$, $\overline{u}=8$ m/s, for the pressure swirl spray, $\overline{D}=17\,\mu m$, $\overline{u}=3$ m/s and for the ultrasonic nebulizer, $\overline{D}=5\,\mu m$, $\overline{u}=1.3$ m/s. Thus the droplet inertia of the aerosol plume is very low. From the multifractal analysis of aerosol plume, we obtain ${H}_{D}=0.51\pm 0.05$, which implies that the data is uncorrelated (Refer Fig. 7) and ${W}_{D}=0.1\pm 0.04$ (close to random noise). Thus, we hypothesize that the droplet inertia is the primary cause of multifractality and the presence of correlations in the droplet diameters.

We also examined the variation of the width of the multifractal spectrum of the droplet axial velocities. However, *W*_*u*_ does not show a clear distinction between the spray characteristics of the ABA, the ultrasonic nebulizer and the PSA (not shown here). From Figs 2 and 4, we observe that *W*_*τ*_ and *W*_*D*_ can be are robust measures to distinguish between the spray characteristics of the PSA, the ABA and the ultrasonic nebulizer, when measurement is made at the edge of the spray at any axial location from the atomizers. In contrast, this difference is not visible in the mean droplet diameters (Fig. 1). Hence, the width of the multifractal spectrum is a stronger measure to validate the empirical models developed for predicting the ABA, the nebulizer and the PSA performance.

#### Quantification of the persistence of the sprays using the Hurst exponent (*H*)

Hurst exponent is a measure of the amount of correlation in the signal. *H* is indicative of the persistence of a signal. A signal is persistent if a large value is more likely to be followed by a large value and a signal is anti-persistent if a large value is more likely to be followed by a small value^39^. For a persistent signal, *H* lies between 0.5 and 1 whereas, for an anti-persistent signal, *H* lies between 0 and 0.5. *H* is 0.5 for an uncorrelated signal such as white noise.

#### The Hurst exponent of multifractal spectrum of the inter-arrival times (*H*_*τ*_)

**Figure 6:**
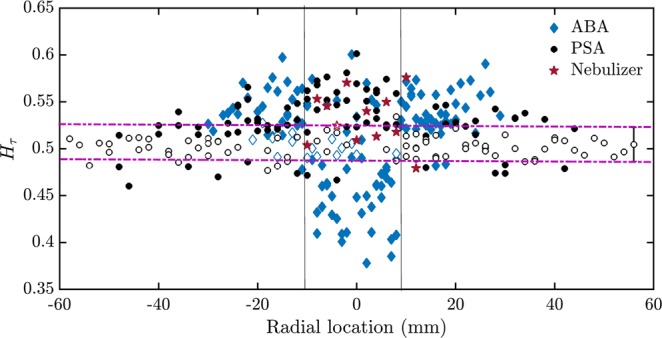
The Hurst exponent of the inter-arrival times of the pressure swirl, the airblast and the ultrasonic nebulizer sprays. Variation of the Hurst exponent of the inter-arrival times (*H*_*τ*_) with radial location of the sprays acquired from the PSA, the ABA and the ultrasonic nebulizer sprays at all measured axial locations. The maximum difference in *H*_*τ*_ of the ABA and the PSA is observed near the spray central axis where inter-arrival times of the droplets from the PSA are persistent while those from the ABA are anti-persistent. *H*_*τ*_ of the ultrasonic nebulizer is almost always persistent. *H*_*τ*_ can be used to determine the locations of droplet clustering and also as a discriminant of the ABA from the PSA and the ultrasonic nebulizer when measurements are obtained near the spray central axis (the region in between the two vertical lines). The horizontal dotted lines show the mean 95% confidence interval around 0.5. The hollow markers indicate the data where *H*_*τ*_ = 0.5 within the confidence interval. The mean error bar is shown on the data point at a radial location of 58 mm.

The persistence in the inter-arrival times encodes information about the clustering of the droplets. The hollow markers in Fig. 6 indicate the cases where no conclusion can be reached as far as persistence is concerned as the inter-arrival times at those radial locations are uncorrelated (*H* = 0.5). The signals corresponding to the filled markers above the purple dotted lines (bounds for the mean confidence interval in which *H* takes a value of 0.5) are persistent (*H*_*τ*_ > 0.5) and those below are anti-persistent behavior (*H*_*τ*_ < 0.5).

We observe from Fig. 6 that near the edge of the spray, the inter-arrival times from the ABA are persistent (long-term positive correlations). Based on the initial fluctuations, if the *τ* between two droplets is small, then the next droplet arrives faster than expected, enabling clustering near the edge of the spray obtained from the ABA. On the other hand, the inter-arrival times of the spray from the PSA are uncorrelated towards the edge of the spray. Alternatively, near the central axis (the region in between the two vertical lines), the *τ*-signal is persistent for the pressure swirl spray and anti-persistent for the airblast spray. The *τ*-signal is almost always persistent for the ultrasonic nebulizer at all the radial locations. Thus, for the spray produced from the PSA, clustering can happen near the spray central axis, for the ABA clustering can happen towards the edge of the spray and for the nebulizer, clustering can happen anywhere.

Surprisingly, near the central axis of the airblast spray, the inter-droplet time signal is anti-persistent (a conclusion that can be drawn with 95% confidence). This indicates that the droplet clustering is not observed in ABA near the spray central axis. However, a mechanism responsible for the presence of this anti-persistent nature is yet to be determined. This is consistent with the experimental study on droplet clustering reported by Henien *et al*.^40^.

Thus *H*_*τ*_ contains information related to the location of droplet clustering in a spray, thereby reducing the need for further analysis using imaging or performing analysis on the histogram of the inter-arrival times. *H*_*τ*_ also shows a distinction in the spray characteristics of PSA, ultrasonic nebulizer and ABA; however, the difference is not as significant as the width of the multifractal spectrum.

#### The Hurst exponent of the multifractal spectrum of the droplet diameter signal (*H*_*D*_)

**Figure 7:**
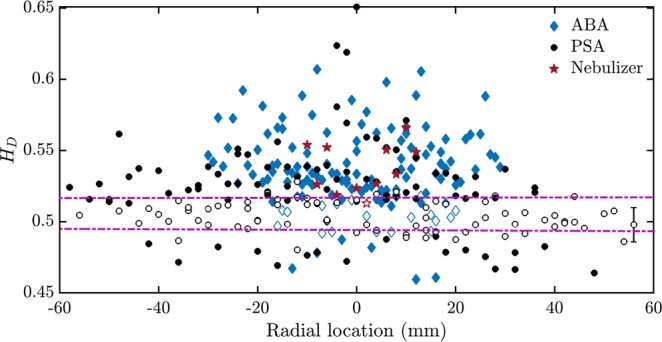
The Hurst exponent of the droplet diameter series of the pressure swirl, the airblast and the ultrasonic nebulizer sprays. Variation of the Hurst exponent of the droplet diameters (*H*_*D*_) with radial location of the sprays acquired from the PSA, the ABA and the nebulizer at all measured axial locations. *H*_*D*_ of pressure swirl sprays are slightly persistent or uncorrelated near the edge of the spray. Near the spray central axis, at most of the radial locations the droplet diameters from the PSA, the ultrasonic nebulizer and the ABA are persistent. The horizontal dotted lines show the mean 95% confidence interval around 0.5. The no-filled markers indicate the data containing *H*_*τ*_ = 0.5 in the confidence interval. The mean error bar is shown on the data point at a radial location of 58 mm.

We observe that near the edge of the spray, the droplet diameter series of the spray obtained from the PSA are mostly uncorrelated or slightly persistent (Fig. 7). Contrarily, those obtained from the ABA and the ultrasonic nebulizer are persistent at almost all radial locations (Fig. 7). In all the sprays, droplet diameter series are persistent near the spray central axis indicating the presence of long-range correlations.

We also studied the Hurst exponents of the axial velocities (*H*_*u*_) of both the sprays. Not surprisingly, the sprays from both ABA, PSA and ultrasonic nebulizer have persistent axial velocities. Classically, this is a well-known characteristic of two-phase turbulent flows^41^. From this study, we observe that *H*_*u*_ cannot be used to distinguish between the sprays from the atomizers.

We observe that all the multifractal measures are scattered in all the figures (Figs 2–7). This is because, we grouped data points corresponding to different axial locations of an atomizer as our objective is to classify the atomizers. The radial locations corresponding to the edge of the spray are different for different axial locations. The other reasons for scatter include turbulence and inherent fluctuations in the experiment.

In addition to the comparison of the multifractal measures across different atomizers, we also compared the variation of multifractal measures for the same atomizer at different Reynolds (*Re*) and Weber (*We*) numbers. We considered the spray characteristics from the PSA at various radial locations at a *Re* of 1030 ± 5 for two *We*, 121.5 and 206. Alternatively, we considered the spray characteristics from the PSA at various radial locations at a *We* of 210 ± 5 for two *Re*, 1034 and 1364. We observed that the variation of multifractal measures, such as *H*_*τ*_ and *W*_*D*_, with radial location, is same irrespective of the flow conditions (Supplementary Figs 1 and 2 shown in Supplementary material). Hence, the multifractal measures do not vary with *We* and *Re* for the same atomizer. We can conjecture that the measures are dependent on the underlying atomization process and hence can be used to distinguish two atomizers.

## Discussion

In this study, we investigated the droplet characteristics of the sprays obtained from the pressure swirl, the airblast atomizers and the ultrasonic nebulizer using MFDFA for the first time. We showed that the Hurst exponent and the width of the multifractal spectrum of the signals of the droplet diameters and the inter-arrival times unveil more information about the complexity in the signals than the standard measures such as mean droplet diameter and mean axial velocity.

We propose that the long range correlations resulting in high widths of multifractal spectrum in the ABA and the ultrasonic nebulizer are due to the strong interaction between the air flow and the droplets. *W*_*τ*_ is an efficient discriminant of the ultrasonic nebulizer from the PSA and the ABA when the measurement is performed near the edge of the spray irrespective of the axial location.

We also note the presence of different long-range correlations in the droplet diameters in the ABA and the ultrasonic nebulizer mainly near the edge of the spray. We propose that these correlations are a result of the presence of vortices in the flow field of the airblast sprays which eject the like-sized particles towards the edge of the sprays. In addition to these, we conjecture that the multifractality in the droplet diameter series is due to the droplet inertia. We report that *W*_*D*_ can distinguish the PSA from the other two atomizers, provided the measurements are made at the edge of the spray. Thus, using *W*_*τ*_ and *W*_*D*_, one can distinguish between the three atomizers when the measurement is performed near the edge of the spray.

We related *H*_*τ*_ to the droplet clustering at various radial locations and we report that it can be used as a measure to identify cluster locations instead of the spatial imaging of the droplets or other statistics. Further, *H*_*D*_ and *W*_*D*_ show the presence of correlation in the droplet diameters in the sprays and hence the droplet diameters cannot be considered as random while modeling the atomization process.

The measures, the Hurst exponent and the width of multifractal spectrum should be used to validate the models in addition to the traditional measures such as droplet size distribution, axial velocity distribution. We showed that MFDFA of the measurements can be used for classifying different classes of atomizers, in addition to improving understanding of atomization process. Thus our analysis shows that the inverse problem of identifying the class of flows from the sparse data is possible using MFDFA. This study can be further extended to classify fluid mechanic systems with similar hardware using a single point measurement in the flow field such as classifying sprays produced during biological process such as breathing, coughing etc. This classification can also aid in developing efficient defense strategies such as classification of submarines based on measurement of velocity from its wake.

## Methods

### Multifractal detrended fluctuation analysis

The brief description of MFDFA is given below.

If the signal is $\{{x}_{k}\}$, where $k=1,2,\ldots \,.,\,N$, and *N* is the length of the signal, a mean subtracted cumulative signal (*Y*) is generated as

$${y}_{k}=\mathop{\sum }\limits_{i=1}^{k}({x}_{i}-\overline{x})$$

where $\overline{x}$is the mean of the signal $\{{x}_{k}\}$

The new signal is then partitioned into *N*_*p*_ non-overlapping windows of equal size *p*. The local trends are detrended using a linear fit $\,\overline{{y}_{i}}$. To account for the scaling of fluctuations at multiple scales in the signal, a structure function $({F}_{p}^{q})$ of order *q* and span *p* is defined as

$${F}_{p}^{q}={(\frac{1}{{N}_{p}}\mathop{\sum }\limits_{i=1}^{{N}_{p}}{(\frac{1}{p}\mathop{\sum }\limits_{k=1}^{p}{({y}_{i}(k)-\overline{{y}_{i}})}^{2})}^{\frac{q}{2}})}^{\frac{1}{q}}$$

The slope of the linear regime in the logarithmic plot of ${F}_{p}^{q}$ versus various span sizes, *p* gives the generalized Hurst exponent *H*^*q*^ of order *q*. The Hurst exponent corresponding to the correlation of the signal is the generalized Hurst exponent of order 2 (*H*^2^ or *H*).

These generalized Hurst exponents can be represented using a spectrum of singularity exponents *f*(*α*) using a Legendre transformation where *α* is a singularity exponent. The Legrendre transform to obtain the singularity spectrum is given as^42^

$$\begin{array}{rcl}{\tau }_{q} & = & q{H}^{q}-1\\ \alpha  & = & \frac{\partial {\tau }_{q}}{\partial q}\\ f(\alpha ) & = & q\alpha -{\tau }_{q}\end{array}$$

The multifractal spectrum is represented as the plot between *f*(*α*) and *α*. The width of the multifractal spectrum is given by $W={\alpha }_{max}-{\alpha }_{min}$. The Hurst exponent provides information related to the long term correlations and memory of a signal. The width and skewness of the multifractal spectrum determine the complexity and multifractality of a signal. For instance, for a monofractal signal, the multifractal spectrum collapses to a single point.

We perform MFDFA on the spray characteristics, the droplet diameters, the inter-arrival times and the axial velocities. The data is divided into segments of 7000 data points each. The multifractal measures such as the width of the multifractal spectrum and the Hurst exponent are computed for each window. To compute the multifractal spectrum for these signals, all possible scales ranging from 16 to 1024 are considered since the data appears to be very noisy. The mean of the values computed for each window is plotted and the standard deviation of these measures gives the error bar shown in the figures representing a 95% confidence interval. We plotted the mean Hurst exponent and the width of the spectrum at each location and the error bar is given by the standard deviation of these measures.

### Experimental method

**Figure 8:**
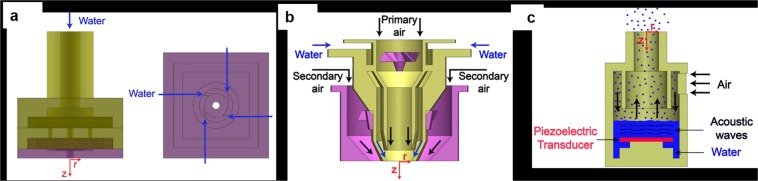
Experimental setup of the PSA, the ABA and the ultrasonic nebulizer. Schematic of the experimental setup of (**a**), the PSA (**b**), the prefilming ABA and (**c**), the ultrasonic nebulizer. The axes *r* and *z* indicate the radial and axial directions, respectively.

The PSA, the ABA and the ultrasonic nebulizer are shown in Fig. 8. In the PSA, water (indicated by blue arrows) enters the atomizer inlet and fills the annulus. The top view of the swirl chamber is shown in Fig. 8a. The liquid enters into the swirl chamber (indicated in pink color) through four tangential inlet slots (the water flow is indicated with blue arrows) and converts the kinetic energy of the liquid into swirl energy. A thin conical swirling liquid sheet exits the final orifice with an air-core formation in the atomizer center. The liquid sheet further disintegrates into drops and forms a hollow cone spray. A pre-filming airblast atomizer is shown in Fig. 8b. The pink color indicates the primary and secondary swirler region of the atomizer. The air and water flows are indicated with black arrows and blue arrows. Initially, the slow-moving thin liquid film is formed in the pre-filmer. Then the primary and the secondary air with high swirl velocity interact with the thin liquid film and forms a spray with fine droplets. The ultrasonic nebulizer is shown in Fig. 8c. The air and water flows are indicated with black and blue arrows, respectively. In the ultrasonic nebulizer, the piezoelectric transducer (shown in red) immersed inside the water, produces the ultrasonic actuation. The acoustic waves generated due to the ultrasonic actuation, travel towards the free surface of the water, and form an aerosol (shown as blue droplets). The air flow is then used to transport the aerosol.

The spray characteristics such as droplet diameter, axial velocity and its inter-droplet arrival times are simultaneously measured using Atrium^®^ Phase Doppler Interferometer (PDI). PDI is a point based measurement technique in which two laser beams of same wavelength, but shifted in frequency, intersect in space. The intersecting region forms the measurement volume. The detector receives a Doppler signal from a drop passing through the measurement volume. The system can measure drop sizes within a range of 0.5 to 1500 μm with an accuracy of 0.5 µm and the velocity values within a range of −100 to 300 m/s with an accuracy of ±0.2%.

The measurement volume is traversed along different radial locations in the mid-plane of spray at different axial locations. At each measurement locations, more than 10,000 samples were captured. The sampling location along the radial direction is equally binned such that the last bin has a minimum sampling rate of at least 10 Hz. For the spray from PSA measurements are performed at the axial locations of 11 mm, 22 mm, 33 mm and 44 mm from the atomizer exit. Similarly for the air blast spray, the measurements are performed at the axial locations are at 9.5 mm, 12.5 mm and 25 mm from the atomizer exit. Finally, for  the nebulizer, the measuments are performed at an axial location of 5 mm from the atomizer exit.

Detailed descriptions of the measurement techniques, experimental setups of the pressure swirl and the airblast atomizers are given in Dhivyaraja *et al*.^34^ and Rayapati *et al*.^35^, respectively.

## Supplementary information

Supplementary Information
