# Supplementary material for: Analysis and classification of droplet characteristics from atomizers using multifractal analysis
Source: Sci Rep. 2019 Nov 7;9:16218. doi: 10.1038/s41598-019-52596-6 (PMC6838191; doi:10.1038/s41598-019-52596-6)
Supplement: Supplementary file 1 — Supplementary Information [file 41598_2019_52596_MOESM1_ESM.pdf]

Analysis and classification of droplet characteristics from atomizers using multifractal analysis – Supplementary Information

V. Godavarthi[1], K. Dhivyaraja[2], R. I. Sujith[1*] and M. V.Panchagnula[2]

[1]Department of Aerospace Engineering, Indian Institute of Technology Madras, Chennai 60036, India.
[2]Department of Applied Mechanics, Indian Institute of Technology Madras, Chennai 600036, India.

*Correspondence and requests for materials should be addressed to R.I.S (sujith@iitm.ac.in)

Variation of multifractal measures of the droplet characteristics at different flow conditions

We compare the performance of multifractal measures H_τ and W_D for a pressure swirl spray at different Reynolds numbers (*Re*) and Weber numbers (*We*). This will aid in strengthening our conclusion that multifractal measures the underlying complexity of atomization process and hence can distinguish different atomizers. Supplementary Fig. S1 shows the variation of **a**, H_τ and **b,** W_D for the PSA across different radial locations corresponding to two different *Re* = 1037, 1364 at a *We* of 210 ± 5, respectively. We observe that there is no significant difference in the variation of multifractal measures.

Further, to compare the effect of change in *We*, we analyzed the droplet characteristics of a PSA at a *Re* of around 1030 ± 5 and *We* of 206, 121.5, respectively. Supplementary Fig. S2 shows the variation of H_τ and W_D for PSA across at different *We*. We observe that there is no significant difference in the variation of multifractal measures. Hence, the multifractal measures do not vary with *We* and *Re* for the same atomizer. We can conjecture that the measures are dependent on the underlying atomization process and hence can be used to distinguish two atomizers.

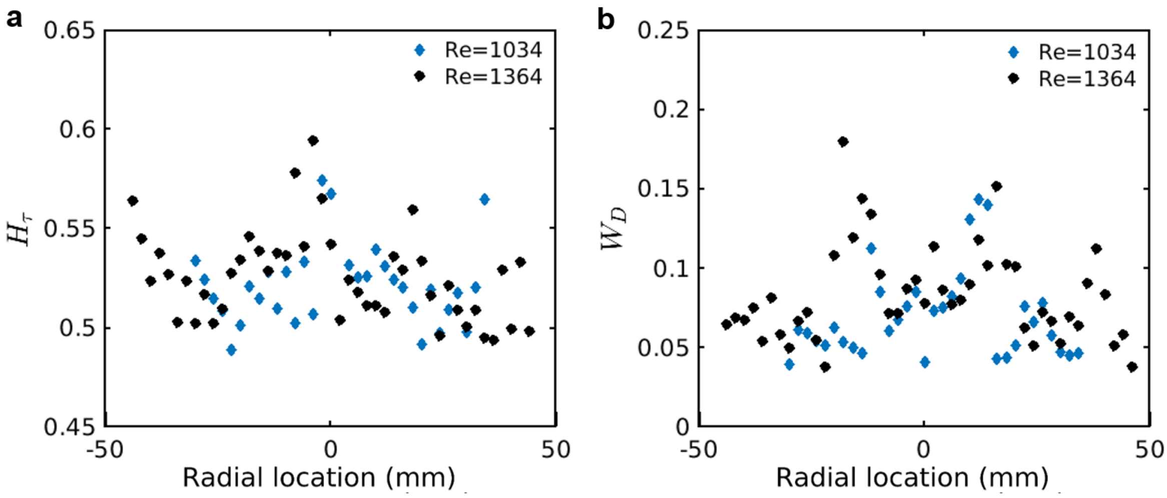

Supplementary Figure S1. Comparison of multifractal measures of the pressure swirl sprays at different *Re*. Variation of **a,** H_τ and **b,** W_D across different radial locations of the sprays obtained from the PSA corresponding to two different *Re* = 1034 and 1364, both at *We* = 210±5. There is no significant difference between the multifractal measures at different Re.

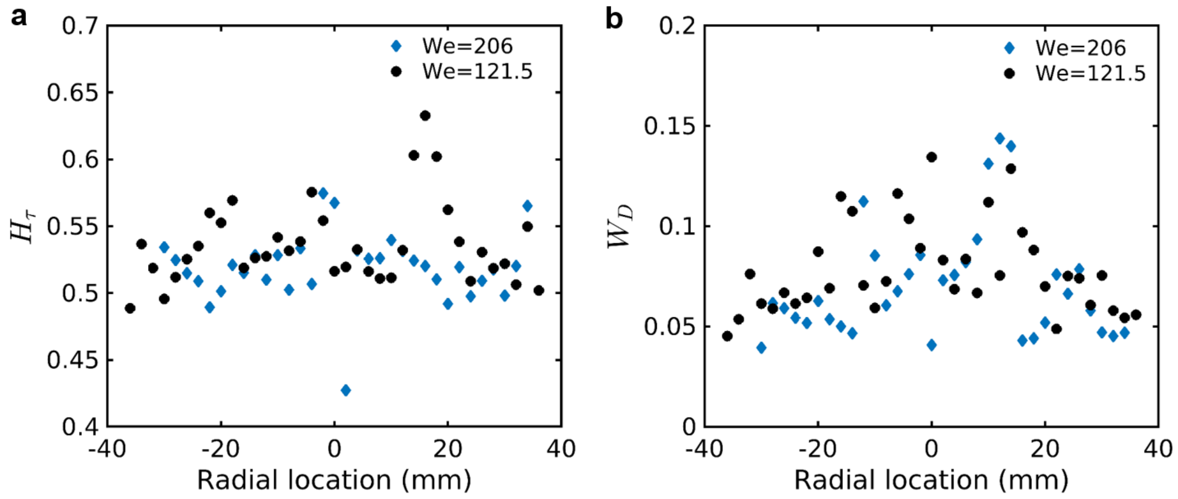

Supplementary Figure S2. Comparison of multifractal measures of the pressure swirl sprays at different *We*. Variation of **a**, H_τ and **b**, W_D across different radial locations of the pressure swirl sprays obtained from the PSA corresponding to two different *We*, 206 and 121.5, both at *Re*=1030±5. There is no significant difference between the multifractal measures at different *We*.

Comparison of droplet diameter distributions of different atomizers

Fig. S3 shows the *pdf* distribution of droplet diameters of pressure swirl, airblast and the ultrasonic nebulizer sprays. We observe a similar left skewed *pdf* distribution for all the atomizers. However, the width of the droplet size distribution of the ultrasonic nebulizer spray is lower than the pressure swirl and the airblast sprays. This is because, ultrasonic nebulizer sprays have much smaller diameter sizes compared to the sprays obtained from the ABA and the PSA.

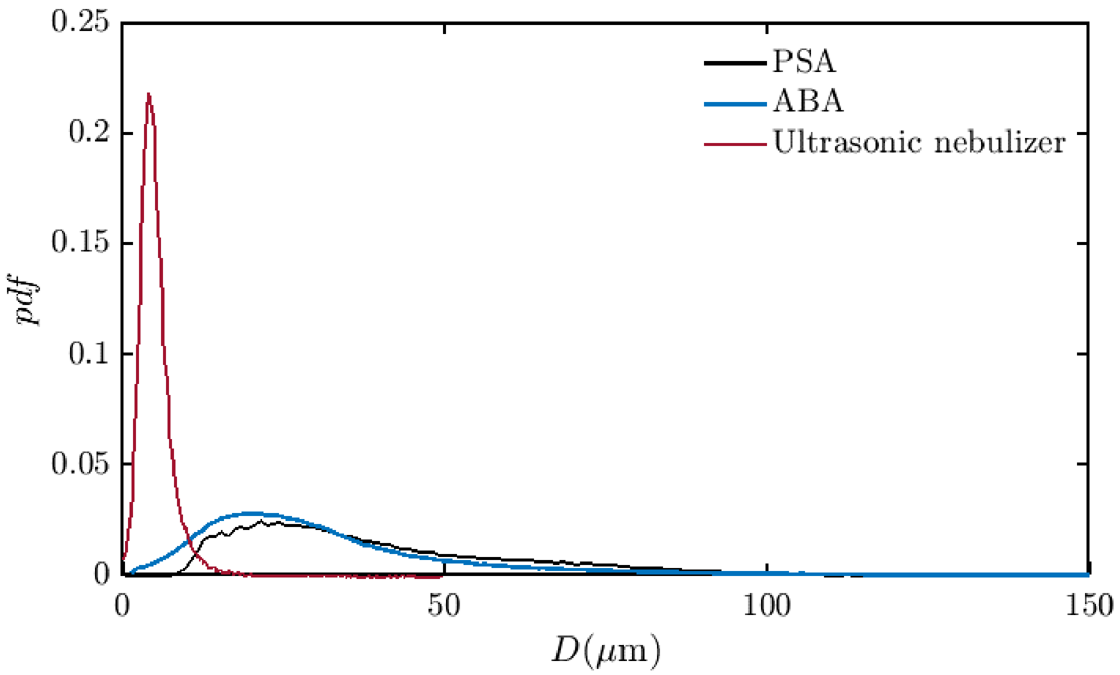

Supplementary Figure S3. The pdf distribution of droplet diameters of pressure swirl, airblast and ultrasonic nebulizer sprays. The *pdf* corresponding to all the sprays are left skewed and cannot be used as a discriminant between the ABA and the PSA.

The consequence of this difference in the width is reflected in the variation of W_D (Figs. 4, 5 in the article). Long range correlations and the broad *pdf* result in the multifractality of a signal. In case of PSA, the long range correlations do not play a major role (shown in Fig.5 in the article). Hence, the broader *pdf* distribution of the droplet sizes of the pressure swirl spray than the airblast spray is the reason for multifractality. Hence, using W_D, we are quantifying the

effect of both the *pdf* distribution and the long range correlations in a signal. However, the *pdf* alone cannot distinguish between the atomizers.
